# Machine Learning Prediction of NO_x_ Emissions from Sewage Sludge Incineration

**DOI:** 10.3390/toxics14100877

**Published:** 2026-09-30

**Authors:** Suwan Wei, Guanghua Wu, Jinli Zhou, Zhigang Jiang, Hong Li, Zhenyi Qian, Ping Lv, Xiaoqian Wang, Efeng Ma, Jun Chu, Junliang Wang, Lian Fan, Min Wu

**Affiliations:** 1 College of Materials Science and Engineering, Zhejiang University of Technology, Hangzhou 310014, China; 2 Shaoxing Research Institute, Zhejiang University of Technology, Shaoxing 312000, China; 3 Zhejiang Zheneng Binhai Environmental Protection Energy Co., Ltd., Shaoxing 312000, China; 4 Zhejiang Carbon Neutral Innovation Institute, Zhejiang University of Technology, Hangzhou 310014, China; 5 International Science and Technology Cooperation Base in Carbon Emission Reduction and Monitoring Authorized by Zhejiang Provincial Department of Science and Technology, Hangzhou 310014, China

**Keywords:** machine learning, NO_x_ emission, sludge incineration, non-catalytic reduction

## Abstract

Accurate prediction of NO_x_ emissions is critical for optimizing selective non-catalytic reduction systems in circulating fluidized bed boilers co-firing sewage sludge and coal. This study develops a data-driven framework by using operational records. A multidimensional feature engineering scheme incorporating auto-regressive, combustion-related, denitration-related features is constructed. Six models—linear regression, random forest, XGBoost, backpropagation neural network, tuned XGBoost, and a stacking ensemble—are systematically compared. SHAP analysis reveals that the model correctly learns the key physicochemical mechanisms, including the temperature window effect and the reducing role of ammonia. The results provide a reliable predictive basis for precise ammonia injection control under fluctuating sludge incineration conditions.

## 1. Introduction

Nitrogen oxides (NO_x_) are major gaseous pollutants from coal-fired industrial boilers. With tightening global emission regulations, precise emission control has become a core challenge for thermal power and waste-co-processing sectors [1,2]. Circulating fluidized bed (CFB) boilers are widely adopted for sludge co-incineration due to their fuel flexibility and uniform temperature distribution. However, frequent fluctuations in sludge blending ratio and load dynamically alter fuel-nitrogen content and temperature fields, complicating NO_x_ formation and exacerbating emission variability; conventional operation struggles to maintain both stable compliance and economic efficiency [3,4,5,6,7,8].

Selective non-catalytic reduction (SNCR) is commonly applied in boilers owing to low capital cost and ease of retrofit. Yet traditional SNCR systems rely on manual experience or Proportional-Integral-Derivative (PID) feedback, which suffer from large lag, insufficient regulation accuracy, high ammonia slip, and reagent waste, often leading to transient exceedances or over-injection under load changes [9]. Existing optimization studies usually fall into the followed categories: CFD-based structural optimization of injectors, reaction-kinetics mechanistic modeling, and data-driven NO_x_ prediction for spray control [10,11,12]. Among these, machine learning methods can build feature-property relationship out of massive data which cannot be analyzed in traditional ways. Ensemble algorithms like random forest and XGBoost have demonstrated superior accuracy over linear regression, and deep learning is increasingly explored [13,14]. Meanwhile, SHAP (SHapley Additive exPlanations) offers an effective tool to interpret black-box models and verify consistency with actual process mechanisms [15,16].

Despite progress, systematic comparisons of multiple algorithms, combined with interpretability analysis and closed-loop control design, remain scarce for complex industrial scenarios with fluctuating sludge co-firing. This study addresses this gap by using the data from sewage sludge incineration. The present results not only provided a trained model which can be used for precise ammonia injection control, but also help us understand the physical mechanism during the sludge incineration processes.

## 2. Methods

**Figure 1:**
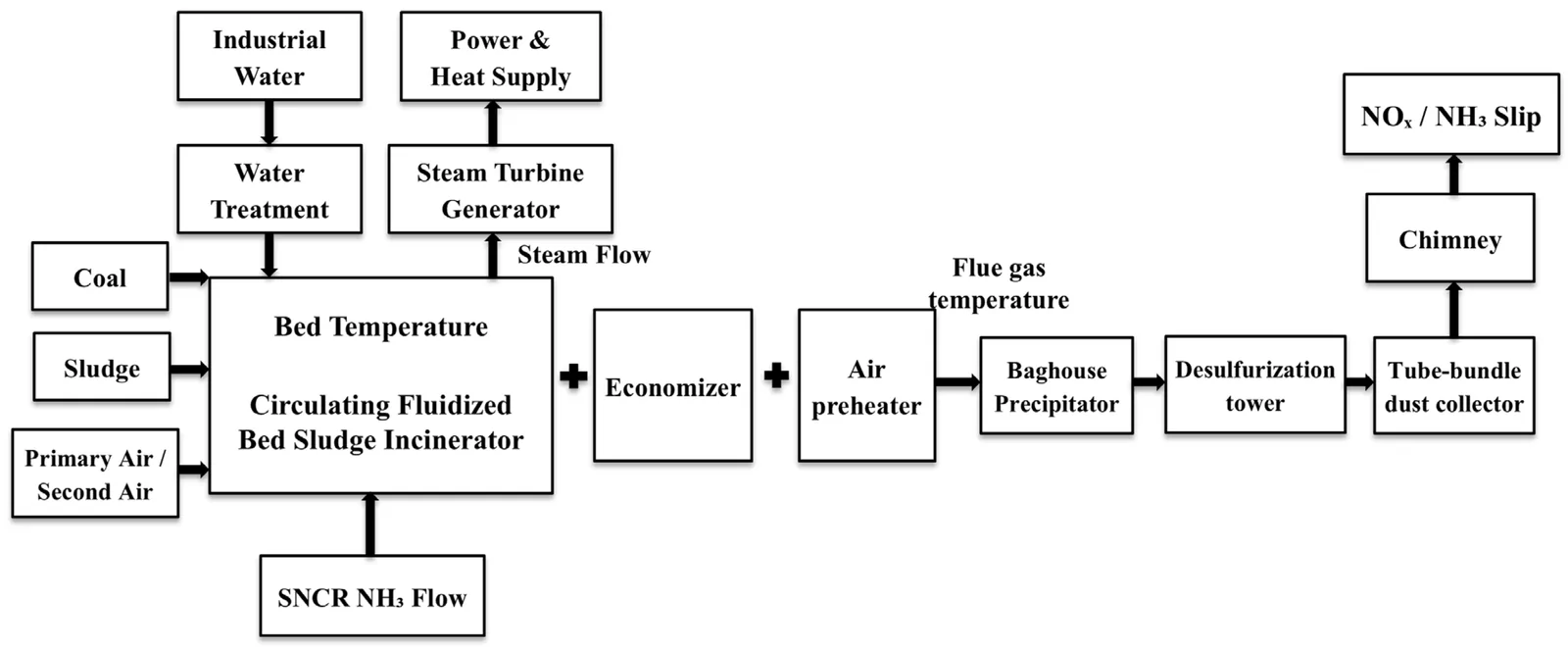
Illustration of the sludge incineration process of the 130 t/h CFB unit. Dewatered sludge is dried and fed, together with coal and with primary and secondary air, into the CFB furnace, where ammonia is injected as the SNCR reagent. The flue gas then passes through the cyclone separator, the economizer, the air preheater, the baghouse precipitator, the desulfurization tower and the tube-bundle dust collector before leaving the stack, where NO_x_ and ammonia slip are monitored. On the steam side, treated water is evaporated in the boiler and the steam is expanded in the turbine generator to supply power and heat.

All the data used in the present work were collected from a printing and dyeing sludge incineration facility located in Zhejiang Province, China. The sludge incineration is carried out in a 130 t/h high-temperature, high-pressure circulating fluidized bed boiler. Aqueous ammonia at a mass fraction of 20% is mixed with dilution water, atomized with compressed air and injected as the SNCR reagent through lances arranged in the horizontal inlet duct of each cyclone separator and at the outlet of each separator. The sludge incineration processes are illustrated in Figure 1, in which the sludge went through drying, incineration, ash disposal and eventual flue gas emission. The facility operates continuously with a distributed control system (DCS) that logs operational parameters at 1 min intervals. The raw dataset comprises 41,085 records recorded in one-month continuous sampling, while the key features includes flue gas emissions of NO_x_, coal feed rate, sludge feed rate, ammonia flow rate, steam temperature, etc.

NO_x_ and NH_3_ slip are monitored by a Fourier-transform infrared analyzer (Zhejiang Zheneng Binhai Environmental Protection Energy Co., Ltd., Shaoxing, China) installed at the 30 m level of the stack, with a measuring range of 0–200 mg/Nm^3^, weekly calibration against a NO standard gas of 83.3 ppm and an expanded uncertainty of 2% of full scale. Throughout this work the target is the O_2_-corrected stack NO_x_ concentration at time t and the most recent input is the sample at t − 5 min, so the prediction horizon is exactly 5 min ahead; lagged inputs are therefore written as t minus the lag. The raw 1 min data were first resampled to 5 min averages to reduce instrument noise and measurement fluctuations, while also matching the approximate 5 min response lag characteristic of the system. The as-fired sludge has an ash content of 57.36% and a lower heating value of 3.68 MJ/kg, while the coal has an ash content of 8.86% and a lower heating value of 21.19 MJ/kg; on a mass basis the blend is about 8% coal and 92% sludge, corresponding to roughly 34% and 66% of the heat input. A total of 56 effective features were constructed covering incineration mechanism, denitrification mechanism and historical results. All features were standardized using Z-score normalization (Standard Scaler), with scaling parameters fitted exclusively on the training set and applied to validation and test sets to prevent data leakage [17]. To rigorously prevent data leakage, which is a critical concern in time-series forecasting, the dataset was split chronologically with a 30 min isolation buffer. In total, 70% of the data was included in the training set, while 15% was used for validation and the rest 15% was used for testing. The 30 min isolation buffer (30 samples of the 1 min record, equivalent to 6 samples after 5 min resampling) between adjacent splits ensures that no future information influences model training, preserving the temporal integrity of the evaluation.

Four representative ML algorithms were selected for comparative evaluation:

Linear Regression (LR) [18], XGBoost [19], Backpropagation Neural Network (BP-NN) [20,21,22,23] and random forest (RF) [24]. Six models were evaluated: four representative algorithms (linear regression, random forest, XGBoost and a backpropagation network), a tuned XGBoost for the SHAP interpretation, and a stacking ensemble. The backpropagation network is a multilayer perceptron (16 and 8 hidden units, ReLU, L2 penalty 0.1, up to 800 iterations, early stopping on a 15% validation fraction; learning rate 0.001 and mini-batch 200 at the scikit-learn defaults). The stacking ensemble combines linear regression and the tuned XGBoost through a linear regression meta-learner fitted on validation-set base predictions. The tuned XGBoost was chosen from 729 hyper-parameter combinations on the validation set. All models share random seed 42; the test split was never used for tuning. SHAP (SHapley Additive exPlanations) was employed to interpret the XGBoost model predictions which provides both global feature importance rankings and local explanations by computing the marginal contribution of each feature to the prediction [15]. This approach enables validation of whether the learned patterns align with established chemical mechanism—specifically, whether ammonia flow rate negatively contributes to NO_x_ (consistent with SNCR), and whether bed temperature positively contributes (reflecting thermal NO_x_ formation). 

## 3. Results and Discussion

### 3.1. Data Analysis

#### 3.1.1. Data Distribution

**Figure 2:**
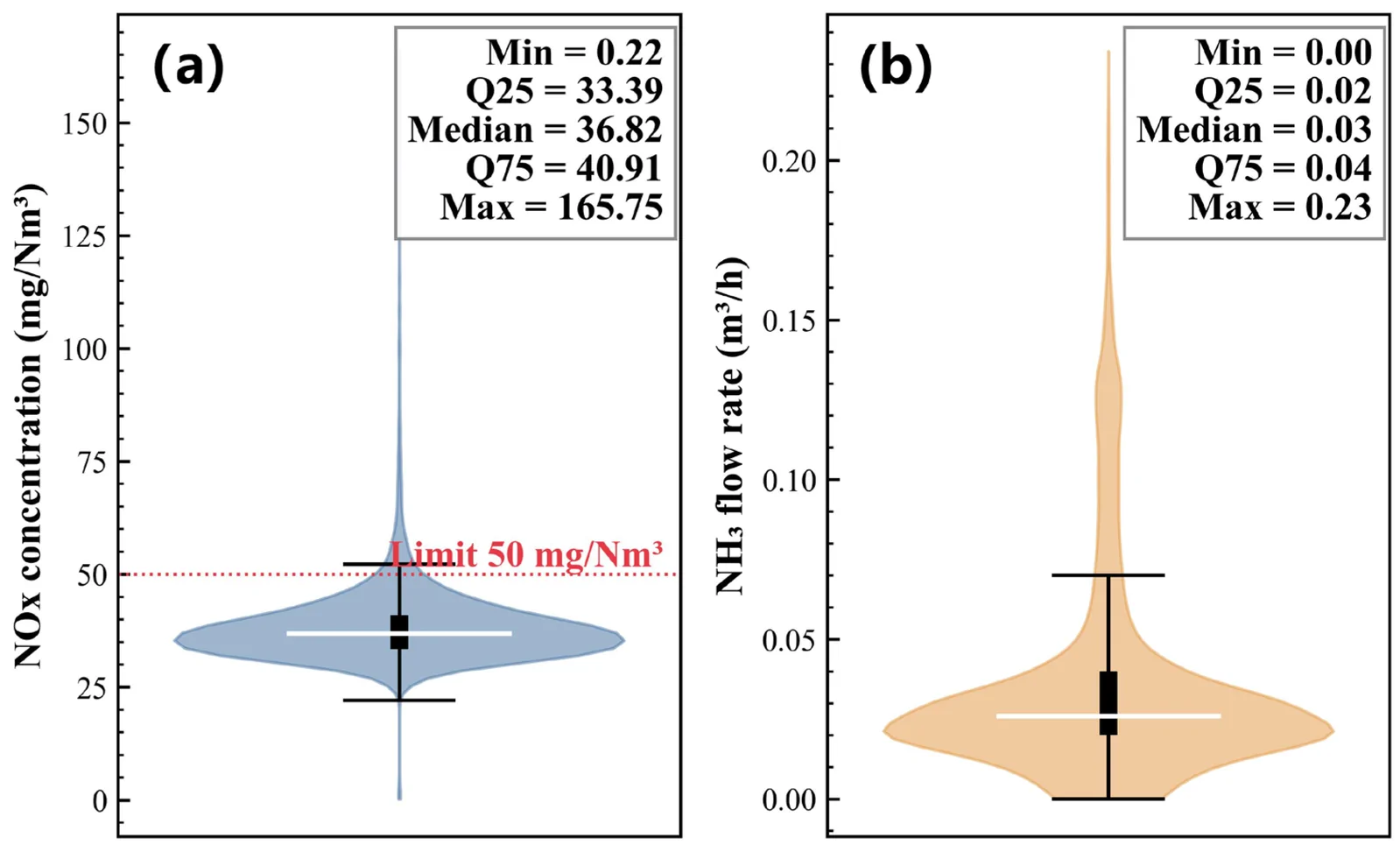
Data distributions of (**a**) NO_x_ concentration and (**b**) aqueous ammonia flow over the full record. The violin outlines the kernel density, the box marks the interquartile range and median, and the whiskers extend to the 5th and 95th percentiles.

Multi-dimensional visualization was performed to characterize raw data distribution. Figure 2 presents violin-box combined plots for two core operational variables: NO_x_ concentration and ammonia flow. NO_x_ concentration mainly distributes within 25–50 mg/Nm^3^ with an average value of 38.34 mg/Nm^3^, generally meeting emission standards. The maximum NO_x_ concentration reaches 169.50 mg/Nm^3^, indicating prominent over-limit spikes corresponding to working condition mutations or startup/shutdown processes. On the other hand, ammonia flow exhibits a wide distribution range, proving great potential to cut ammonia consumption and operational costs via process parameter optimization.

#### 3.1.2. Data Processing

Industrial DCS data commonly contain anomalies caused by sensor faults, over-range readings and negative values, which severely disrupt model training. All core variables were assigned upper and lower physical bounds according to process principles and equipment rated ranges. Values exceeding thresholds were marked as missing and later filled via time-series interpolation [25]. Physical boundaries were defined following three rules: (1) compliance with basic physical significance (non-negative flow and concentration); (2) matching equipment rated measuring ranges; and (3) covering all normal working conditions while only removing extreme instrument failure data [26]. The cleaned statistics of the processed data are given in Table 1. The zero entries in Table 1 are kept rather than discarded because they mark distinct operating states: the ten one-minute zero readings of NO_x_ occur as isolated single-minute points while the boiler was at full load and correspond to analyzer zeroing, the 451 zero readings of main steam flow form a single 7.5 h block during which the furnace remained in normal combustion and indicate a dropout of the calculated flow signal, and the 2849 zero readings of aqueous ammonia flow correspond to periods in which no reagent was being dosed.

### 3.2. Feature Engineering

#### 3.2.1. Feature Extraction

To fully capture dynamic characteristics of boiler combustion and SNCR denitrification, features including the incineration and denitration related variables were extracted, including the ammonia flow, bed temperature, coal volume, sludge volume, etc. More importantly, since the eventual NO_x_ emission is highly time-series related, the lagged features up to 30 min were also constructed to capture the physical delays.

#### 3.2.2. Pearson Correlation Coefficient

**Figure 3:**
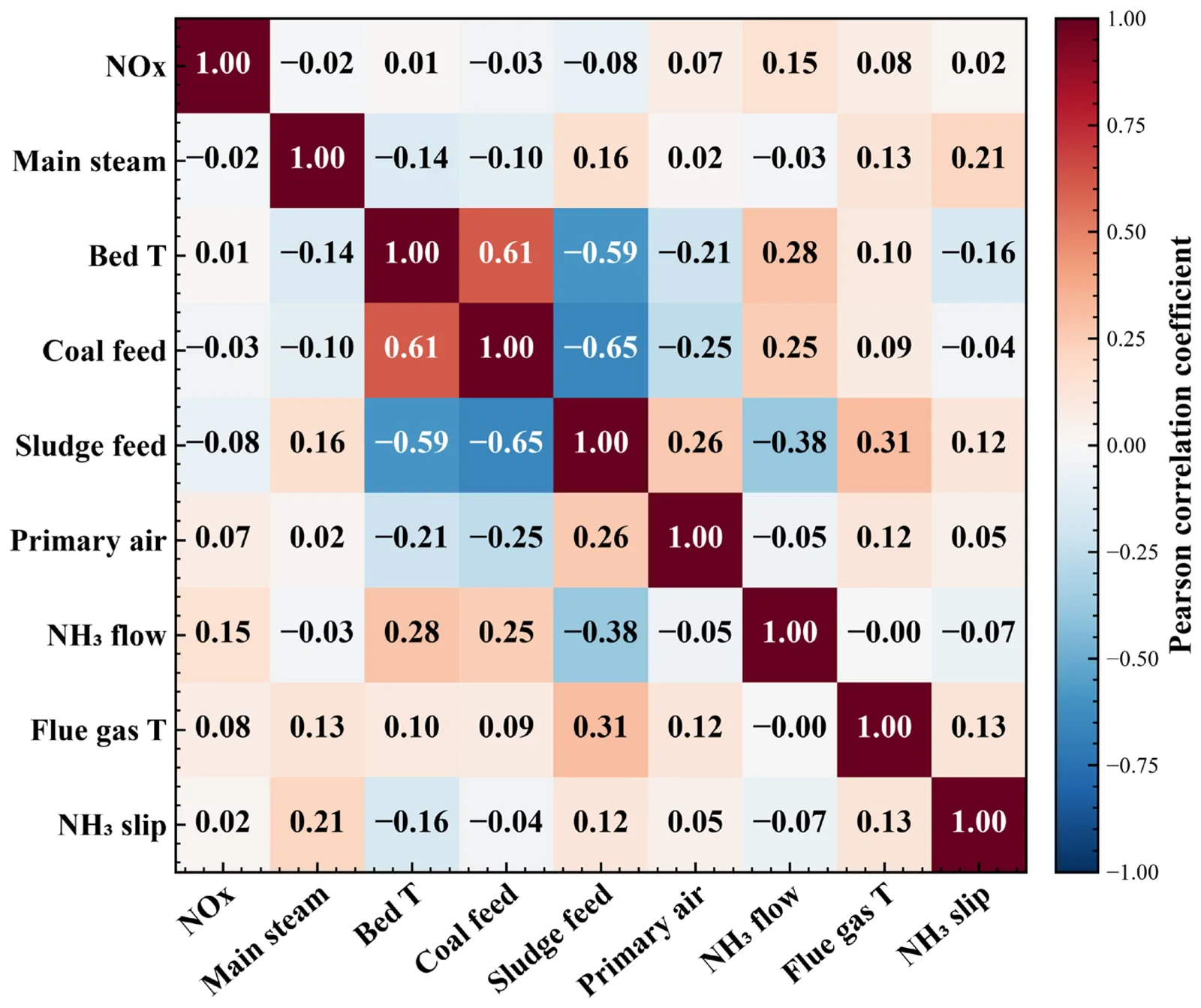
Pearson correlation heatmap of the core features (5-min averages).

Pearson correlation coefficients of core features were calculated and visualized via heatmap (Figure 3). The correlation coefficient between instantaneous NO_x_ and ammonia flow equals approximately 0.15, presenting weak positive correlation. This phenomenon does not contradict denitrification reaction mechanisms but represents typical statistical features of industrial closed-loop feedback systems-operators usually increase ammonia injection when NO_x_ rises, forming the superficial “high NO_x_ matching high ammonia dosage” pattern [27]. This result indicates that precise prediction cannot rely on instantaneous process variables alone, and that the historical evolution of the process must be represented to capture the operating condition transmission [28]. As expected, the coal volume stimulate the incineration and it positively correlates with bed temperature, while sludge volume negatively correlates with bed temperature.

#### 3.2.3. Feature Importance Screening

A two-step screening method combining variance threshold filtering and random-forest importance ranking based on the mean decrease in variance was used to remove redundant features: low-variance invalid features were eliminated first, then features with a low mean decrease in variance were discarded, leaving 56 core features for modeling [24]. The 56 features comprise six historical NO_x_ values (5–30 min), 48 historical values of eight operating parameters (bed temperature, flue-gas temperature, main steam flow, coal feed rate, sludge feed rate, primary and secondary air flow, aqueous ammonia flow and ammonia slip, each at 5–30 min), and two derived ratios (unit-load ammonia dosage and sludge blending ratio). StandardScaler Z-score normalization was implemented to eliminate dimensional differences and accelerate convergence of neural networks and linear models [18]. Normalization parameters were fitted only on the training set and reused for validation and test subsets to strictly avoid data leakage.

**Figure 4:**
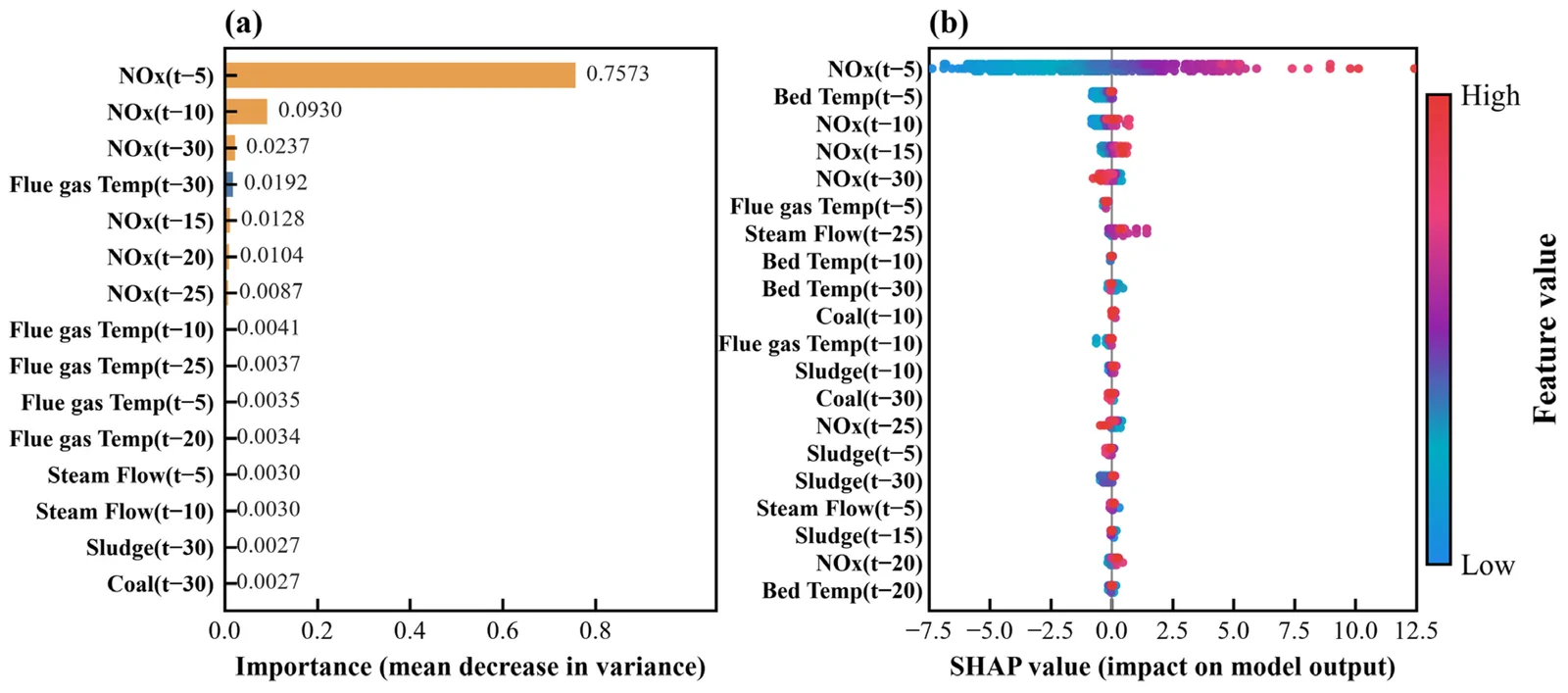
(**a**) Random-forest feature importance, expressed as the mean decrease in variance; (**b**) global SHAP importance. Both panels use the same feature order, so they correspond row by row. In (**b**), points are coloured by the corresponding feature value (red, high; blue, low).

Feature importance analysis identifies NO_x_(t − 5) lag term as the dominant predictor, far exceeding all other variables (Figure 4a). Here t − 5 represents the feature adopted 5 min earlier. The feature importance from the SHAP analysis gave the same picture (Figure 4b). This results from strong physical inertia of industrial combustion-denitrification systems rather than data leakage: physically, continuous combustion processes create robust temporal autocorrelation of NO_x_; algorithmically, tree models prioritize this stable inertial pattern. Time-series hard splitting and isolation band design exclude future information leakage [17].

### 3.3. Training Results of Machine Learning Models

**Figure 5:**
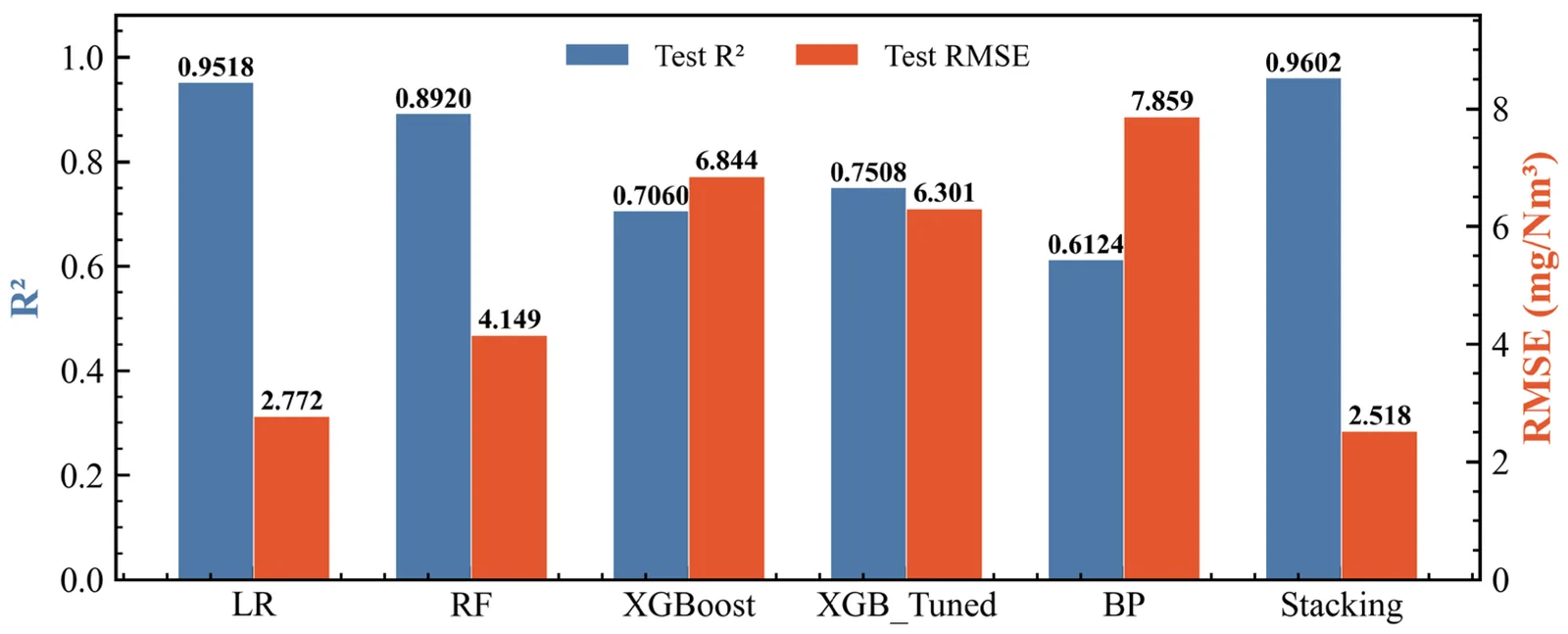
Performance comparison of the six models on the training and test sets.

**Figure 6:**
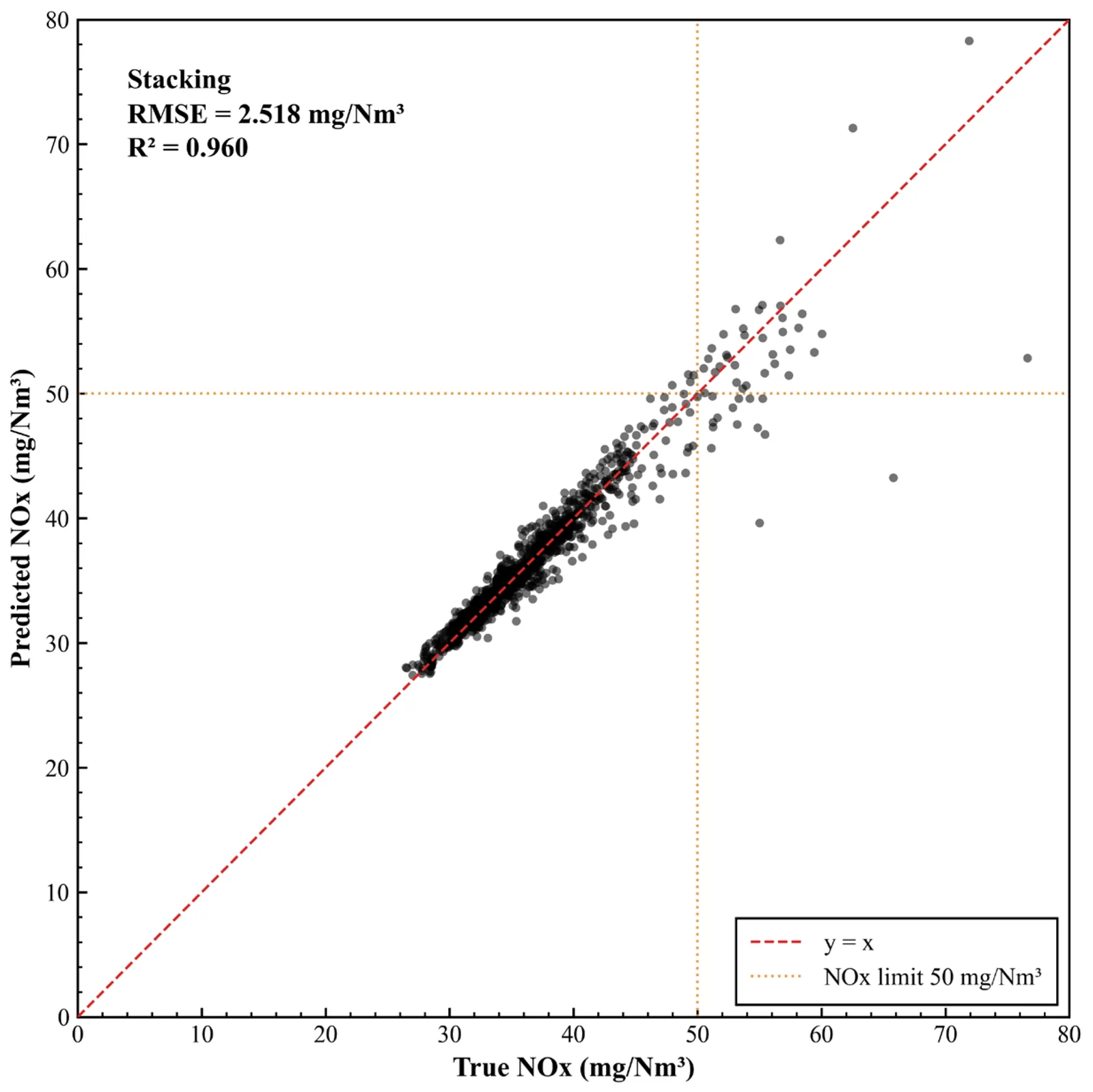
Predicted against observed NO_x_ for the stacking model on the test set; the dashed line is the 1:1 line.

**Figure 7:**
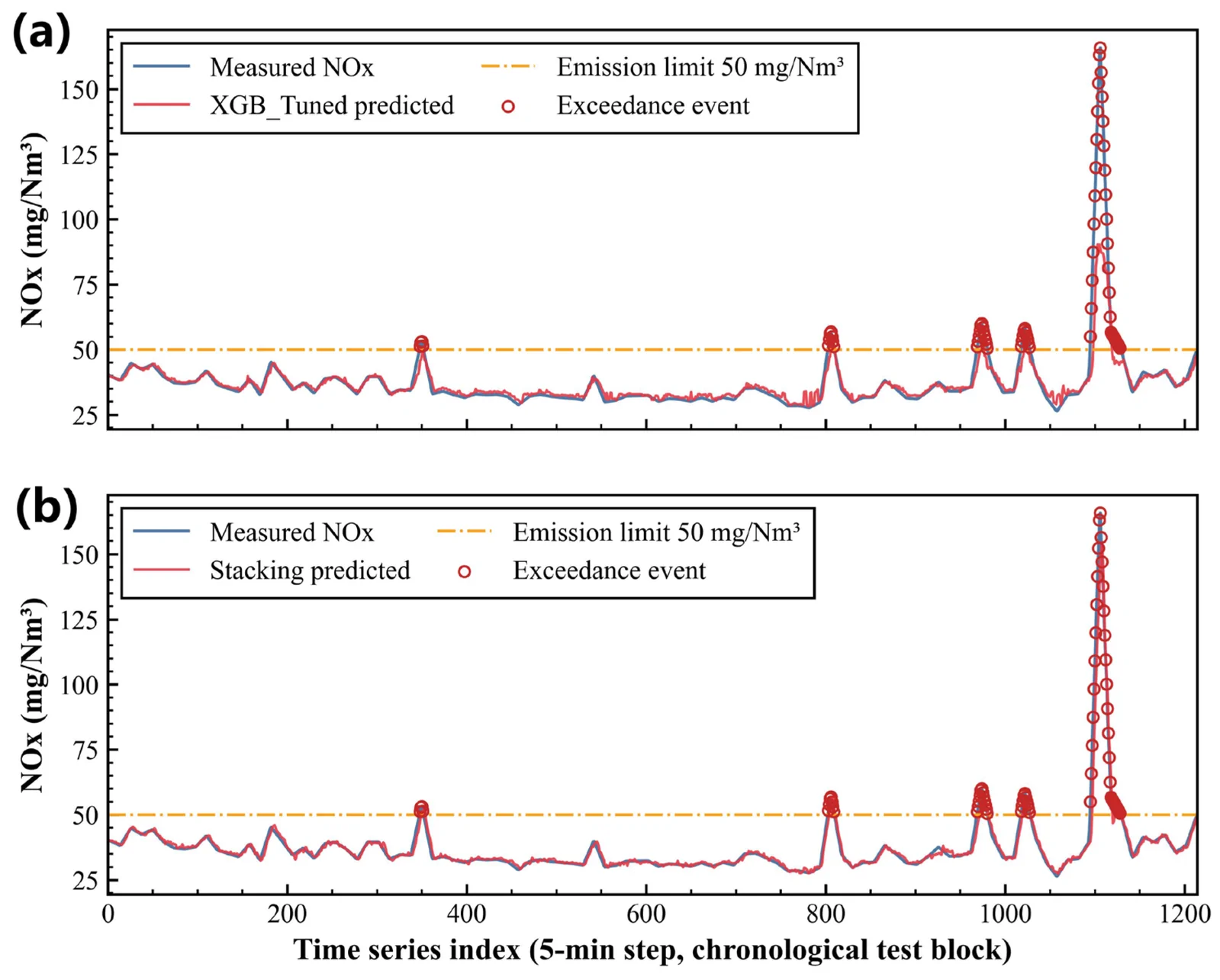
Time-series prediction on the test set: (**a**) tuned XGBoost model; (**b**) stacking ensemble. The dashed line marks the 50 mg/Nm^3^ emission limit; the exceedance events are captured by both models.

Figure 5 compares R^2^ and RMSE metrics of six models on training and test sets, leading to the following key findings: (1) The BPNN model gives the worst performance among these selected models, with a R^2^ value of only 0.6124. The reason is a capacity limitation rather than a training artifact: the fixed 16 × 8 architecture has to fit 5745 samples in 56 dimensions, whereas the dominant information in NO_x_ is carried by the lagged terms and therefore requires almost no nonlinear mapping. (2) The LR model surprisingly performed quite well with a Test R^2^ value of 0.9518, probably due to the dominant feature of NO_x_(t − 5) as discussed above. (3) The unoptimized XGBoost model attains ultra-high training accuracy yet drastically drops in test performance, indicating a certain degree of overfitting. Regularization tuning and early stopping effectively mitigate overfitting and elevate test accuracy, verifying generalization improvement via tree regularization [19,29,30]. (4) The Stacking model provides the best performance, with the highest test R^2^ (0.9602) and lowest RMSE (2.518 mg/Nm^3^). The good prediction performance of the stacking model can be found in the scatter point plot as shown in Figure 6. The superior performance against the XGBoost model can be found in Figure 7. The “linear baseline + nonlinear compensation” stacking strategy with improved model performance is consistent with the previous studies [13,31].

### 3.4. SHAP Dependence Analysis

SHAP (SHapley Additive exPlanations) [15] was applied to conduct global and local interpretability analysis on tuned XGBoost. Derived from game-theoretic Shapley values, SHAP quantifies individual feature contributions to single-sample predictions and shows whether the model relies on predictive associations that are consistent with process knowledge rather than on data noise. TreeExplainer was adopted for efficient and precise computation on tree-based models [15]. This method has been widely deployed in denitrification modeling to decode internal black-box decision logic and support data-driven process optimization [16,32].

**Figure 8:**
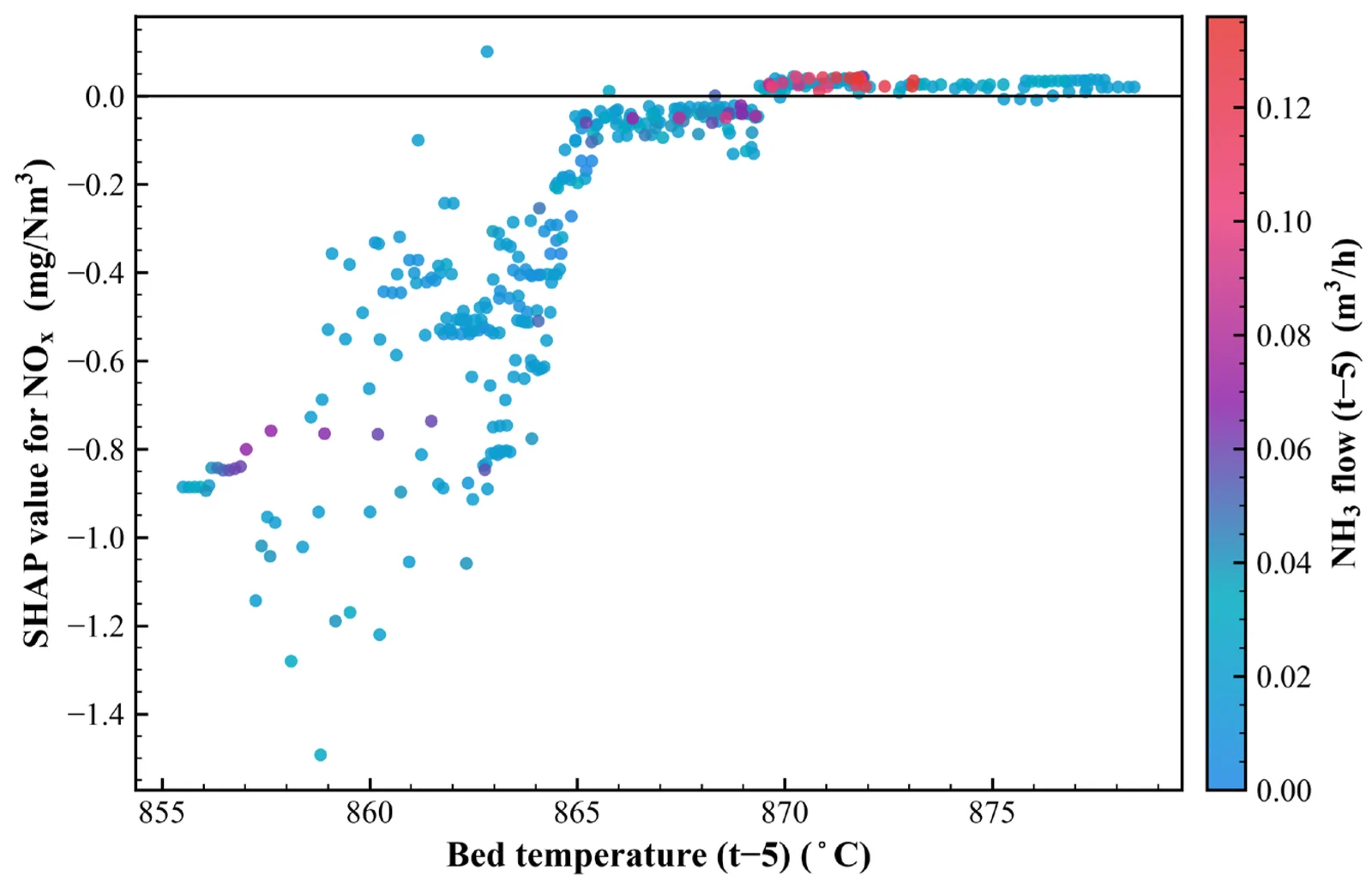
SHAP dependence of bed temperature at t − 5 min, colored by the aqueous ammonia flow. The shaded interval marks the 860–865 °C region; the temperature dependence shown is a statistical association, not a chemical optimum.

Figure 8 illustrates SHAP dependence of bed temperature, colored by ammonia flow to analyze coupled effects of these two key features. Apparently, bed temperature exerts substantial influence on SHAP values. In the low temperature range (855–860 °C), maximum ammonia dosage fails to deliver strongest negative inhibition, consistent with the SNCR temperature window: reduction activity is insufficient at low temperatures and limits denitrification efficiency [1,11]. In the temperature range of 860–865 °C, the bed temperature generates strong negative NO_x_ suppression, yet inhibitory strength decays with rising temperature as combustion oxidation accelerates NO_x_ formation. In the temperature range of 865–870 °C, balanced NO_x_ levels emerge where ammonia reduction and combustion oxidation counteract each other. While for temperature above 870 °C, SHAP values turn positive, indicating ammonia reduction is overwhelmed by NO_x_-promoting combustion oxidation. The coupling relationship between ammonia dosage and bed temperature provides theoretical support for precise ammonia injection. Targeted ammonia dosing within the optimal temperature window simultaneously reduces emissions and operational costs.

## 4. Conclusions

This study develops a machine learning framework for predicting NO_x_ emissions from a sludge-co-fired CFB boiler. Among six models, the stacking ensemble (LR + tuned XGBoost) yields the highest accuracy (R^2^ = 0.9602, RMSE = 2.518 mg/Nm^3^), outperforming individual models. SHAP analysis identifies predictive associations indicating that the model learns physically consistent mechanisms: ammonia reduces NO_x_, sludge increases NO_x_, and a distinct temperature window (≈860–865 °C) governs SNCR efficiency. These findings enable reliable ahead-of-time predictions and provide a quantitative basis for graded ammonia injection strategies. Prospective control evaluation is needed to establish actual reagent savings and compliance. The proposed framework demonstrates practical potential for intelligent denitration control under fluctuating co-firing conditions, and its transfer to other boilers with similar operational challenges would require independent validation on external data, since the present results come from a single unit.

## Figures and Tables

**Table 1:** Presents the descriptive statistics of key operational features.

Feature	Average	Min.	Median	Max.
NO_x_ (mg/Nm^3^)	38.34	0.00	36.76	169.50
Ammonia flow (m^3^/h)	0.03	0.00	0.03	0.24
Flue gas T (°C)	440.97	300.25	448.17	512.52
Ammonia slip (ppm)	0.11	0.10	0.11	0.12
Main steam flow (t/h)	129.02	0.00	131.21	147.66
Bed temperature (°C)	876.62	845.02	875.97	943.96
Primary air flow rate (Nm^3^/h)	65,595.91	61,739.43	65,624.97	69,532.84
Secondary air flow rate (Nm^3^/h)	41,849.85	0.00	42,873.29	76,743.05
Coal feed rate (t/h)	4.81	1.68	4.77	11.45
Sludge feed rate (t/h)	53.75	0.04	55.58	75.28

## Data Availability

The raw data supporting the conclusions of this article will be made available by authors on request.
